# Supplementary material for: Splice-Junction-Based Mapping of Alternative Isoforms in the Human Proteome
Source: Cell Rep. Author manuscript; Available in PMC 2020 Jan 15. (PMC6961840; doi:10.1016/j.celrep.2019.11.026)
Supplement: 3 [file NIHMS1546469-supplement-3.zip › DF2/PXD009737/TestisLysC-98-Q96PE3-KHTSSGCQSIIYIPQDVVRAK.pdf]

A

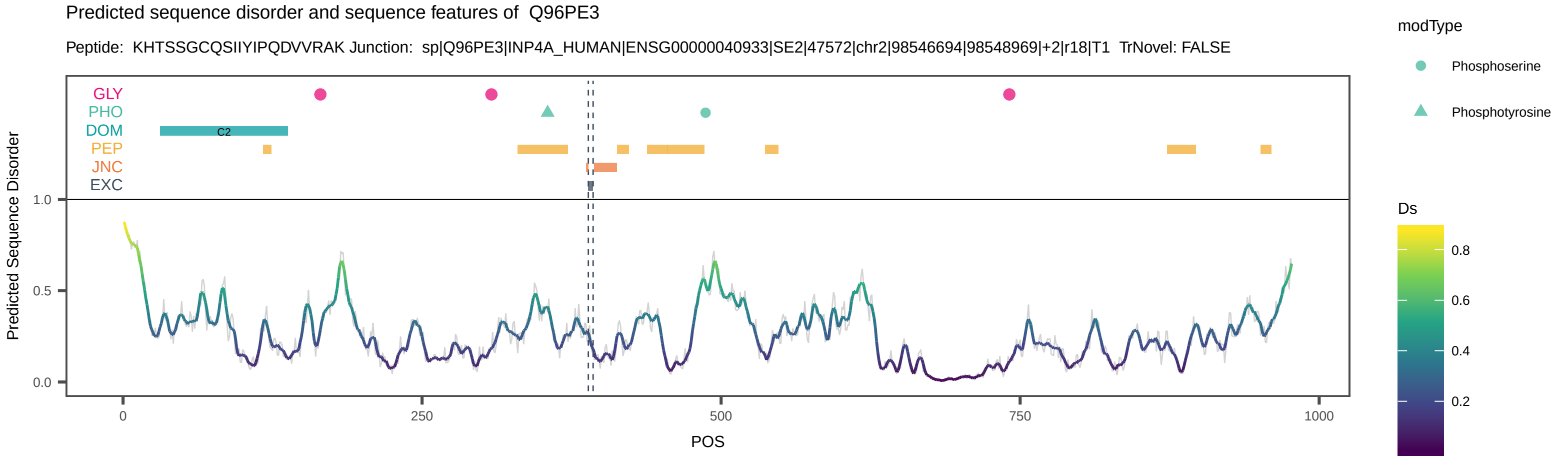

Predicted sequence disorder and sequence features of Q96PE3
Peptide: KHTSSGCQSIIYIPQDVVRAK Junction: sp|Q96PE3|INP4A_HUMAN|ENSG00000040933|SE2|47572|chr2|98546694|98548969|+2|r18|T1 TrNovel: FALSE
GLY
PHO
DOM
C2
PEP
JNC
EXC
Predicted Sequence Disorder
1.0
0.5
0.0
0
250
500
750
1000
POS
modType
Phosphoserine
Phosphotyrosine
Ds
0.8
0.6
0.4
0.2

B

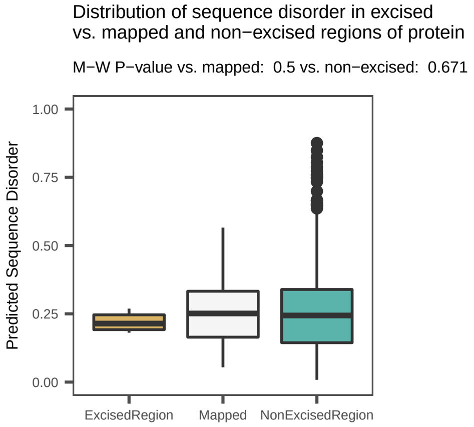

Distribution of sequence disorder in excised
vs. mapped and non−excised regions of protein
M−W P−value vs. mapped: 0.5 vs. non−excised: 0.671
Predicted Sequence Disorder
1.00
0.75
0.50
0.25
0.00
ExcisedRegion
Mapped
NonExcisedRegion

C

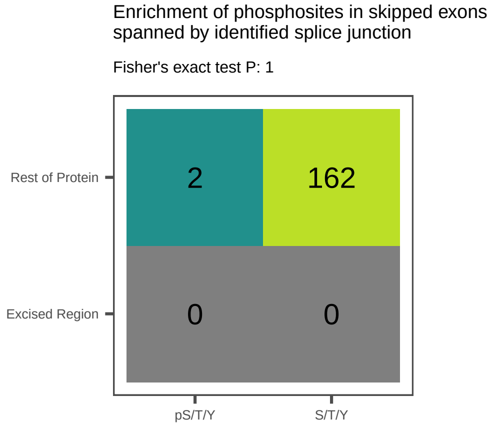

Enrichment of phosphosites in skipped exons
spanned by identified splice junction
Fisher's exact test P: 1
Rest of Protein
Excised Region
2
162
0
0
pS/T/Y
S/T/Y
